# Management of infertility in women with hypothalamic hypogonadotropic hypogonadism: an expert opinion

**DOI:** 10.1186/s12958-026-01535-y

**Published:** 2026-02-19

**Authors:** Geoffroy Robin, Lorraine Maitrot-Mantelet, Sophie Dubourdieu, Bérengère Kiehl-Bigot, Maria Katsogiannou, Michel De Vos, Sophie Christin-Maitre

**Affiliations:** 1 Department of Endocrine Gynaecology, CHU Lille, Lille University, Lille, France; 2 Department of Gynecology Obstetrics and Reproductive Medicine, Paris Center University Hospital/Cochin, Paris, France; 3 Reproductive Medicine, Atlantic Polyclinic, Nantes, France; 4 Ferring SAS, Gentilly, France; 5 ICTA PM, Fontaine les Dijon, France; 6 Brussels IVF, Universitair Ziekenhuis Brussel, Brussels, Belgium; 7 Department of Endocrine and Reproductive Medicine, Center of Endocrine Rare Diseases of Growth and Development (CRESCENDO), FIRENDO, Endo- ERN, Hospital Saint-Antoine, Paris, France

**Keywords:** Pulsatile GnRH therapy, Hypogonadotropic hypogonadism, Congenital hypogonadotropic hypogonadism, Functional hypothalamic amenorrhea, Polycystic ovarian morphology

## Abstract

**Background:**

Hypothalamic gonadotropin-releasing hormone (GnRH) plays a central role in regulating the pituitary-gonadal axis. The pulsatility of GnRH release is critical for maintaining the function of GnRH receptors and the secretion pattern of gonadotropins, namely follicle-stimulating hormone (FSH) and luteinizing hormone (LH), which regulate endocrine function and follicular growth and maturation. During the luteal phase, LH is crucial for supporting a functional corpus luteum and stimulating it to produce progesterone, estradiol and relaxin.Hypothalamic hypogonadotropic hypogonadism originates from a deficiency in GnRH secretion. Low circulating gonadotropin levels subsequently lead to reduced ovarian function and anovulation. This condition may be congenital or acquired, for example through functional hypothalamic amenorrhoea (FHA) or FHA combined with polycystic ovarian morphology (PCOM). Pulsatile GnRH therapy plays a pivotal role in restoring the physiological menstrual cycle and selecting a dominant follicle in these women, thereby inducing ovulation and achieving fertility. There is extensive literature accounting for a high ovulation rate and consequently high pregnancy and birth rates per cycle, with a lower risk of adverse outcomes.

**Results:**

In this review, based on clinical evidence and published studies, we provide recommendations for the alternative treatment of infertility in women with congenital hypothalamic hypogonadotropic hypogonadism (CHH) and FHA (with or without PCOM), until pulsatile GnRH therapy becomes available again or in countries where this device is not marketed. Starting doses and adjustments should be made according to the aetiology of hypothalamic hypogonadotropic hypogonadism and other patient parameters. In all cases, luteal phase support is imperative and should ideally be provided by hCG injections to optimize corpus luteum functions.

**Conclusion:**

When pulsatile GnRH therapy is not available, and to ensure the effective treatment of female infertility due to FHA (with or without PCOS) or hypothalamic CHH, we advise physicians to optimise stimulation with exogenous gonadotropins according to the cause of hypothalamic hypogonadotropic hypogonadism. In all cases, providing luteal phase support by optimising corpus luteum function is mandatory.

## Introduction

### Gonadotropins and ovarian physiology

#### Hypothalamic regulation of gonadotropins

Pituitary gonadotropin secretion is regulated at the hypothalamic level: pulsatile secretion (approximately every 90 min during the follicular phase) of hypothalamic gonadotropin-releasing hormone (GnRH) induces gonadotropin biosynthesis and secretion by binding to specific membrane receptors on pituitary gonadotropin cells. Regulation of the activity of GnRH neurons (located in the arcuate nucleus of the hypothalamus) is a complex phenomenon involving other neurons such as Kiss neurons (which secrete kisspeptin) and KNDy neurons (which secrete Kisspeptin - Neurokinin - Dynorphin) [1].

#### Follicular phase: the ‘two cells - two gonadotropins” bicellular theory

**Fig. 1:**
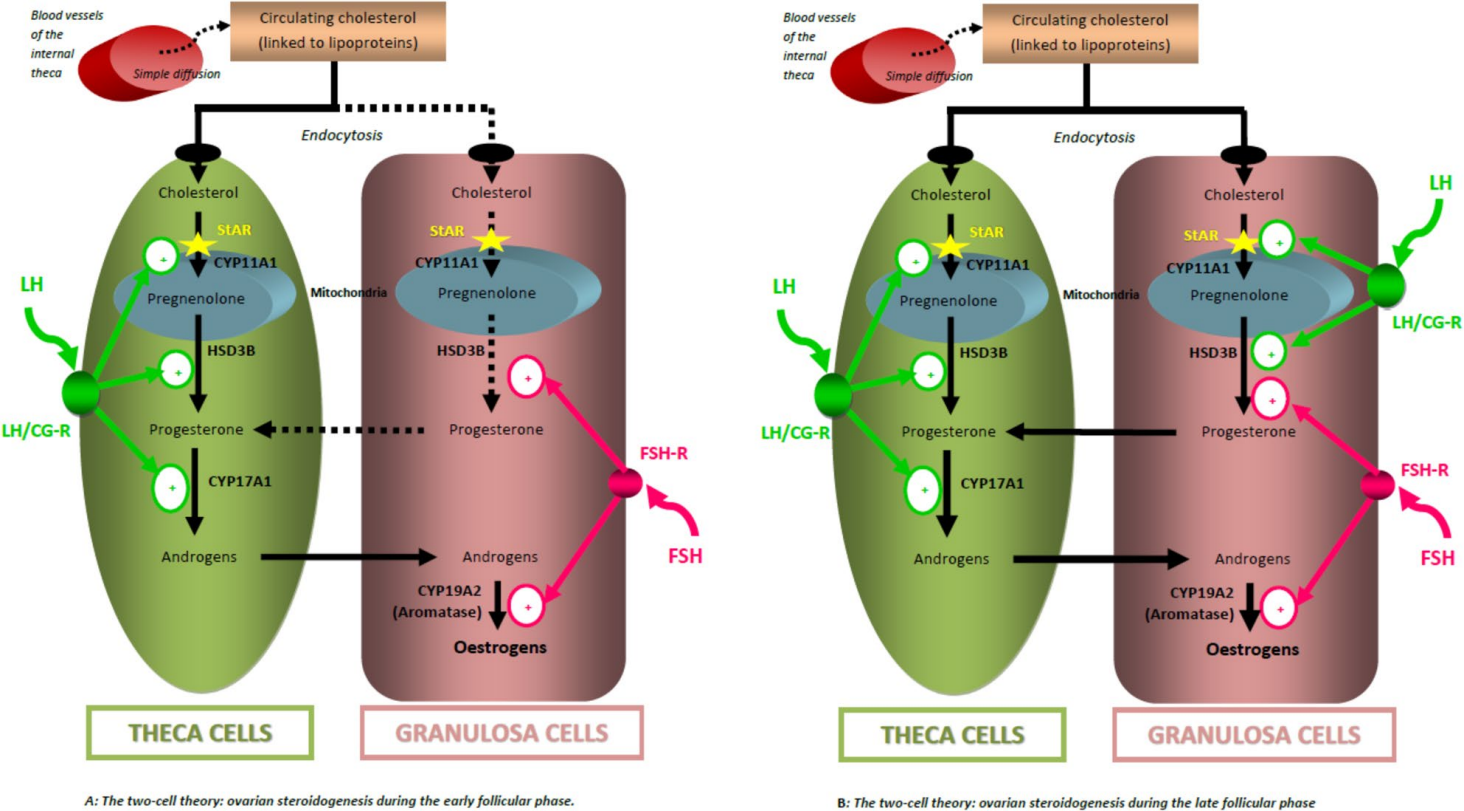
Bicellular theory during the follicular phase. FSH: Follicle Stimulating Hormone; LH: Luteinizing Hormone; StAR: Steroidogenic Acute Regulatory protein; CYP11A1: Cytochrome P450 11A1ou P450 SCC: Cytochrome P450 Side Chain Cleavage enzyme; LH/CG-R: Luteinizing Hormone/choriogonadotropin Receptor; HSD3B: 3β-Hydroxysteroid Dehydrogenase; FSH-R: Follicle Stimulating Hormone Receptor; CYP17A1: Cytochrome P450 17A1 with both 17α-hydroxylase and 17–20 lyase activities; CYP19A2: Cytochrome P450 19A2 or aromatase

The bicellular theory of the ovary is essential for understanding steroid hormone production during the follicular phase of the menstrual cycle, which corresponds to ‘gonadotropin-dependent’ cyclical follicular recruitment (2nd phase of folliculogenesis preceding ovulation) (Fig. 1). During the early follicular phase, the secretion of luteinizing hormone (LH) by the gonadotropic cells of the pituitary gland stimulates the production of androgens. FSH (follicle-stimulating hormone) is produced by the same cells as LH and stimulates the granulosa cells to convert the androgens produced by the theca cells into estrogens (aromatase activity) [2–4]. FSH has been demonstrated to stimulate the proliferation of granulosa cells, thereby facilitating follicular growth (Fig. 1a). During the late follicular phase, the granulosa cells also express membrane receptors R-LH/CG (common membrane receptor to both LH and hCG (human choriogonadotropin)). Thus, during this period, LH will act in synergy with FSH and reinforce its action on these same cells in the dominant follicle allowing for adequate proliferation and functional maturation of granulosa cells, followed by full follicular and oocyte maturation [2, 5]. This interaction between theca cells and granulosa cells is crucial for follicular growth and maturation (Fig. 1b).

Some authors have defined the concept of the “LH window”. This corresponds to an interval of LH concentration that allows optimal follicular development: too low a level compromises steroidogenesis and follicular growth, while too high a level could lead to follicular atresia or premature follicular luteinization. Therefore, minimal LH activity is essential to ensure growth and selection of the dominant follicle [5].

#### The pituitary LH : a pivotal element in the luteal phase

According to more recent data, gonadotropins also play a ‘facilitating’ role during initial follicular recruitment (1st phase of folliculogenesis), even though their presence is not theoretically essential to the process [6, 7].

It has been established that during the luteal phase, the progesterone secreted by the corpus luteum exerts a negative feedback control at the hypothalamic level, which leads to a decrease in the frequency of GnRH pulses : from 1 pulse every 90 min during the follicular phase and 1 pulse every 60 min in the immediate pre-ovulatory period, to 1 pulse every 180 to 240 min during the luteal phase [8]. Given its short half-life, LH secretion follows the frequency of pulsatile hypothalamic GnRH. Nevertheless, this hormone will play a crucial role during the luteal phase in the human species [9]. Thus, LH plays a critical role in maintaining a functional corpus luteum during the luteal phase; absent pulsatile stimulation by LH, the corpus luteum undergoes premature atresia. LH also directly stimulates the corpus luteum to produce progesterone and estradiol, as well as other factors such as relaxin (a hormone that plays an important role in embryo implantation and uterine muscle relaxation) [9–11].

### Hypothalamic hypogonadotropic hypogonadism definition

Hypothalamic hypogonadotropic hypogonadism is a condition that originates from a deficiency in the secretion of GnRH. Low circulating gonadotropin levels subsequently lead to reduced ovarian function and anovulation [12]. This condition can be acquired or congenital [12] (causes are summarised in Table 1).

**Table 1:** Hypothalamic causes of anovulation *(From Balen et al. Hum reprod update 2024)*

Causes	Detail
Genetic	• Congenital absence/deficiency in GnRH neurons and their control due to gene mutations
Autoimmune	• Langerhans cell histiocytosis
Iatrogenic	• Pharmacological (sex steroids, GnRH agonists/antagonists, opioïds…) • Radiation • Surgery
Neoplastic	• Craniopharyngioma • Optico-hypothalamic glioma • Germinoma • Metastatic lesions
Functional	• Functional hypothalamic amenorrhea (FHA) due to energetic deficiency (weight loss and/or excessive physical activity) and/or excessive stress • Obesity • Chronic inflammatory illness • Constitutional delay of puberty
Infectious and inflammatory	• Sarcoidosis • Tuberculosis
Trauma and/or vascular	• Head injury
Physiological	• Pregnancy • Breastfeeding
Endocrine	• Thyroid dysfunctions • Hyperandrogenism (not PCOS) • Cushing syndrome • Hyperprolactinemia (both hypothalamic and pituitary anovulation)
Idiopathic	• Idiopathic

The acquired and most common form is functional hypothalamic amenorrhea (FHA). FHA is caused by significant fat and energy restrictions and/or excessive exercise and/or severe emotional stress and is usually reversible [13, 14]. FHA is characterized by a decrease in GnRH pulsatility, leading to reduced amplitude and frequency of LH pulses. This, in turn, can contribute to anovulation [13, 14]. Interestingly, a subgroup of 41.9% − 46.7% of women with FHA present polycystic ovarian morphology (PCOM) on ultrasound scan, according to a recent review from Ott et al. [15]. Because the frequent combination of FHA and PCOM in young women may falsely lead to a diagnosis of polycystic ovarian syndrome (PCOS), a proper understanding of the diagnostic, hormonal, metabolic and therapeutic aspects of this specific variant of FHA is mandatory [15]. Nevertheless, a substantial proportion of these patients with FHA and PCOM will shift towards a proper diagnosis of PCOS after weight gain and restoration of hypothalamic function.

In contrast, the hypothalamic congenital hypogonadotropic hypogonadism (CHH) is a rare, and heterogeneous condition due to a variety of genetic mutations affecting the development or physiology of the hypothalamus [16]. Hypothalamic CHH may be isolated or associated with certain specific symptoms, such as anosmia in the Kallmann-de Morsier syndrome [17].

### Pivotal role of pulsatile GnRH therapy for infertile women with hypothalamic amenorrhea

Pulsatile GnRH administration has been used since 1986; It was approved in several countries such as such as France, Germany, Switzerland, Austria, Canada and the Netherland. It is recognized as a safe and effective treatment for infertility due to hypothalamic hypogonadism [18–22]. This therapy has been developed to restore physiological menstrual cycle, selection of a dominant follicle, thereby inducing ovulation and achieving fertility among women suffering from FHA and hypothalamic CHH. There is a myriad of published studies showing that this treatment results in a high ovulation rate (75.6% − 85.2%) and consequently high pregnancy and birth rates (over 21% and 35.0%, respectively) per cycle (described in meta-analysis [21]). In a recent cohort study, pulsatile GnRH therapy has been shown to be as effective in FHA as in hypothalamic CHH, despite the significantly longer duration of pulsatile GnRH administration in the latter group (respectively 18.16 (± 7.66) days versus 23.59 (± 8.02) ; *p*<.001) [20]. The high efficacy of this treatment in FHA has been corroborated by a 25-year cohort study on pulsatile GnRH therapy, which demonstrated optimal pregnancy rates (80.5%) and low multiple gestation rates (1.6%) per treatment [22]. In agreement with the above studies, pulsatile GnRH therapy is recommended as a first-line treatment for women with FHA [23, 24] and infertility as it seems to be superior to conventional exogenous gonadotropin in achieving ovulation with a lower risk of adverse outcomes [21, 25]. Published data comparing therapeutic aspects of pulsatile GnRH treatment in patients with FHA-PCOM and those with FHA-non-PCOM are scarce. While the use of pulsatile GnRH treatment seems to be equally effective in both groups [26], pulsatile GnRH therapy may be more efficient than exogenous gonadotropins in FHA-PCOM [15, 27, 28] (as well as in FHA-non-PCOM patients [21, 25]).

All previously mentioned studies [20–22, 26] strongly support that the widespread implementation of pulsatile GnRH administration would be advantageous for patients with hypothalamic CHH and FHA, and access to pulsatile GnRH treatment should be promoted in countries where it is not currently available. Indeed, while simple, efficient and safe, pulsatile GnRH administration is relatively underused, since it is only currently accessible in a few European countries, more specifically France, Germany, Austria, Switzerland and the Netherlands. Therapeutic pulsatile GnRH (gonadorelin acetate; Ferring SAS) is provided by LutreLef 3,2 mg administered with LutrePulse™ system, a drug delivery injection device consisting of a disposable LUTREPULSE “pod” and a LUTREPULSE “manager”. The GnRH pump is FDA approved but not yet commercially available in the USA. This may change in view of emerging evidence from a recently completed USA-based multicenter, double-blind, randomized, placebo-controlled trial evaluating three different doses of pulsatile GnRH administered via a subcutaneous pump for ovulation induction in patients with FHA (ClinicalTrials.gov Identifier: NCT01976728).

While increasing evidence calls for more extensive availability and use of pulsatile GnRH therapy, temporary shortage of LutrePulse™ has been announced by Ferring because of the interruption of its manufacturing. Efforts are focused on the development of a new device ; due to the complexity of the administration device development, Ferring has anticipated a period of shortage for approximately 36 to 48 months.

When pulsatile GnRH therapy is not available, effective infertility treatment of women with CHH, FHA, including FHA-PCOM, who desire to conceive, should be guaranteed. We provide, herein below, recommendations for alternatively treating these women.

### Management of infertility in hypothalamic hypogonadotropic hypogonadism when pulsatile GnRH therapy is not available

In cases of hypothalamic hypogonadotropic hypogonadism, patients not receiving any hormonal treatment experience hypo-estrogenic amenorrhea (responsible for endometrial atrophy and sometimes even uterine hypotrophy). In theory, induction of ovulation could be started at any time in these amenorrhoeic women. However, some teams prescribe sequential hormone replacement therapy for at least 1 month, before starting treatment with exogenous gonadotropins. The aim of this practice is to obtain satisfactory uterine and endometrial trophicity. Despite the lack of published data about this is practice, we recommend prescribing such treatment when patients have been deprived from exogenous estrogen supply for several months (considering parity and uterine dimensions on ultrasound).

Given the pathophysiology of hypothalamic hypogonadism and the involvement of the two gonadotropins during folliculogenesis, it will be necessary to use exogenous gonadotropins by using FSH combined with LH activity (either in the form of highly purified hMG (human menopausal gonadotropins) or in the form of a combination of r-hFSH (recombinant human follicle-stimulating hormone) together with r-hLH (recombinant human luteinizing hormone)). There are very few studies comparing these two gonadotropins, whose efficacy appears to be similar in this indication. (reviewed in [29])

The management of infertility in these women also provides an opportunity to review the potential harmful effects of estrogen deficiency on bone and cardiovascular health, particularly in women who have not received optimal estrogen replacement therapy [29].

#### Management of infertility treatment in women with hypothalamic congenital hypogonadotropic hypogonadism (CHH) 

**Fig. 2:**
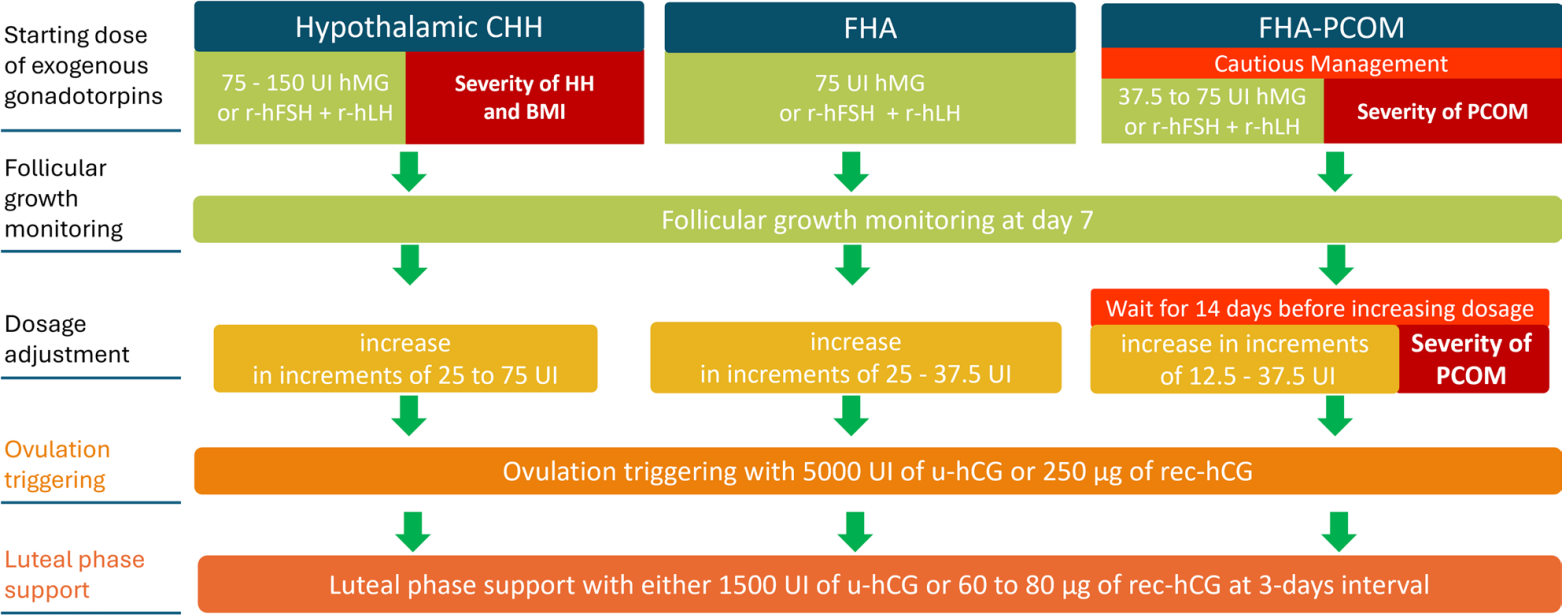
Management of patients with hypothalamic hypogonadotropic hypogonadism when pulsatile GnRH therapy is unavailable. CHH, congenital hypogonadotropic hypogonadism; FHA, functional hypothalamic amenorrhea; PCOM, polycystic ovarian morphology ; hMG, human menopausal gonadotropin; rec-hFSH, recombinant human follicle-stimulating hormone; rec-hLH, recombinant human luteinizing hormone; u-hCG or rec-hCG, urinary or recombinant human chorionic gonadotropin

As previously demonstrated, fertility can be efficiently restored by both pulsatile GnRH therapy and exogenous gonadotropin administration. For these patients, we recommend initiating stimulation with 75 to 150 UI per day of hMG or r-hFSH together with r-hLH [30]. The initial dosage should be modulated according to the severity of the gonadotropic deficiency (the lower the LH and oestradiol, the more severe the gonadotropic deficit) and the patient’s BMI. With regard to this last parameter, there are no data specific to hypothalamic hypogonadism, but by extrapolation to the data concerning the volume of distribution and efficacy of gonadotropins, it is probable that the higher the BMI, the higher the starting dose should be [31]. As previously described by Bry-Gauillard et al. [32], levels of serum AMH (Anti-Mullerian Hormone) and/or antral follicle count (AFC) should not be considered in the choice of dosage because these markers are often lowered in cases of severe hypothalamic CHH without having any deleterious impact on the response to stimulation and live birth rates. Monitoring follicular growth on the seventh day will allow for adjustment of the dosage, if necessary, with increases of 25 to 75 UI/day. It is essential to inform patients that the 1st cycle of exogenous gonadotropin treatment may require a long time. Ovulation triggering with subcutaneous (or intramuscular) injection of 5000 UI of urinary hCG or 250 µg of recombinant hCG (Eq. 6500 UI of urinary hCG) is necessary in these women. A luteal phase support must be proposed to these women, as progesterone production by corpus luteum during the luteal phase is dependant of LH secretion (Fig. 2).

#### Management of infertility treatment in women with FHA without PCOM

We recommend initiating stimulation with 75 UI/day of hMG or r-hFSH together with r-hLH in women with FHA and monitoring follicular growth at day 7 of the treatment. If the desired outcome is not achieved with the initial dosage, it may be increased in increments of 25 to 37.5 UI/day. Ovulation triggering with u-hCG or r-hCG and luteal phase support must be proposed to these women (Fig. 2).

#### Management of infertility treatment in women with FHA-PCOM

Caution is required for the management of patients with FHA-PCOM in order to avoid multifollicular recruitment and multiple pregnancies [15]. We recommend initiating stimulation with lower starting dose of gonadotropins: 37.5 to 75 UI/day of hMG or r-hFSH together with r-hLH, according to the severity of PCOM. Monitoring follicular growth is suggested at day 7 of gonadotropin therapy. As women with PCOS, it would be advisable to apply the rules of the ‘step-up low-dose’ protocol, ensuring a first step of 14 days before increasing the dosage of exogenous gonadotropins (for review [33]). In fact, it is not always possible to determine whether there is an underlying PCOS in these subgroup of FHA women [15]. If needed, the daily dose of exogenous gonadotropin can be adjusted in 12.5 to 37.5 UI/day increments, based on the severity of the PCOM. Sometimes the duration of treatment during the 1st cycle can be a little long. Ovulation triggering with u-hCG or r-hCG and luteal phase support must be proposed to these women (Fig. 2).

#### The importance of luteal phase support

All women suffering from hypogonadotropic hypogonadism (regardless of the underlying cause) have low or no endogenous LH. Therefore, the corpus luteum is insufficiently stimulated by LH during the luteal phase [29, 30]. Some physicians believe, despite lack of evidence, that only progesterone (+/- estradiol) supplementation is able to compensate for luteal phase deficiency. The importance of a functional corpus luteum to limit the risk of vasculoplacental pathologies (such as preeclampsia) has recently been demonstrated in endometrial preparation during frozen-thawed embryo transfer [34]. In addition to secreting progesterone and estradiol, the human corpus luteum is the primary source of various factors that play a crucial role in reproduction. These factors include for example relaxin, which plays an important role during embryo implantation [10, 11]. It is therefore mandatory that, at the end of gonadotropins treatment, these patients should receive subcutaneous (or intramuscular) injection of 1500 IU of urinary-hCG or 60 to 80 µg of recombinant hCG (eq of 1560 to 2080 u-hCG), at a 3-days interval (the 1st injection is usually given five days after ovulation is triggered i.e. three days after the day of ovulation) [29, 30]. In the event of pregnancy, trophoblastic hCG then takes over to stimulate the corpus luteum during the 1st trimester. Given the half-life of hCG, it is important to recommend that patients should not have a blood or urine pregnancy test within 5 days of the last hCG injection.

## Conclusion

Pulsatile GnRH administration has emerged as the treatment of choice for women with FHA (with or without PCOM) or hypothalamic CHH willing to conceive, presenting excellent ovulation, pregnancy and live birth rates and low rates of multiple gestation. When pulsatile GnRH therapy is not available, physicians should optimize stimulations with exogenous gonadotropins. Starting dose of gonadotropins and modalities of dosage adjustments should be adapted according to the etiology of hypothalamic hypogonadotropic hypogonadism. In the event of hypothalamic hypogonadotropic hypogonadism, luteal phase support is imperative and should ideally be provided by hCG injections to optimize corpus luteum functions.

## Data Availability

No datasets were generated or analysed during the current study.
